# Bats Broaden Sonar Field of View to Maneuver around Obstacles

**DOI:** 10.1371/journal.pbio.1001147

**Published:** 2011-09-13

**Authors:** Janelle Weaver

**Affiliations:** Freelance Science Writer, Glenwood Springs, Colorado, United States of America

**Figure pbio-1001147-g001:**
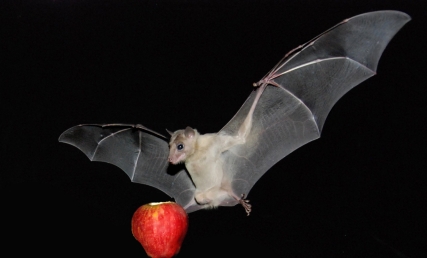
An Egyptian fruit bat landing on an apple in the lab. A recent *PLoS Biology* study shows that the echolocation ability of these bats is much more sophisticated than previously thought.

[Fig pbio-1001147-g001]Spotting a friend in a crowded room is a challenge, but we manage to do so by adjusting our visual spotlight of attention to search our surroundings. Animals that depend on other senses can adapt to similar situations. Egyptian fruit bats (*Rousettus aegyptiacus*), for example, use echolocation to orient inside their caves and to find fruit hidden in the branches of trees in Africa and the Middle East. Their high-frequency emissions form a sonar beam that spreads across a fan-shaped area, and the returning echoes allow them to locate and identify objects in that region. But these bats emit clicks using the tongue rather than the vocal cords and were considered to have little control over their vocalizations, so scientists have puzzled over how they are able to navigate through complex environments.

This month in *PLoS Biology*, a team led by neurobiologists Nachum Ulanovsky and Cynthia Moss reports that Egyptian fruit bats adapt to environmental complexity using two tactics. First, they alter the width of their sonar beam, similar to the way humans adjust their spotlight of attention. Second, they modify the intensity of their emissions. These novel findings show that the echolocation abilities of this species are much more sophisticated than previously thought.

The Egyptian fruit bat emits a pair of successive clicks to inspect a spatial region, aiming the first beam toward the left and the second beam toward the right, or vice versa. The average direction of the two partially overlapping beams typically points toward the item of interest. When multiple objects are present, these bats might broaden their field of view by increasing the angle separating the two sequential beams.

To test this possibility, Ulanovsky and his team trained five Egyptian fruit bats to locate and land on a mango-sized plastic sphere placed in various locations in a large, dark room equipped with an array of 20 microphones that recorded vocalizations. In one set of experiments, the researchers simulated an obstacle-filled forest by surrounding the sphere with two nets spread between four poles. To reach the target, the bats flew through a narrow corridor whose width and orientation varied from trial to trial.

The area covered by each click pair was about three times larger in the mazelike setting compared with the empty room, and the angle separating the beams was about 12 degrees wider. This difference was apparent by the time the bats zeroed in on the target, and it became more pronounced as they entered the corridor. The larger field of view could allow the animals to simultaneously track the sphere and the poles and avoid collisions while landing.

The bats also increased the loudness of their vocalizations by about nine decibels in the presence of obstacles, as if to ensure that enough energy would be distributed among all of the items for them to perceive their surroundings.

The ability of a bat species to change the angle between consecutive beams in a click pair has never before been reported, and it may be unique to Egyptian fruit bats because of their rapid tongue movements. Together, the findings reveal two new strategies bats use to scan multiple objects in complex environments.

**Yovel Y, Falk B, Moss CF, Ulanovsky N (2011) Active Control of Acoustic Field-of-View in a Biosonar System. doi:10.1371/journal.pbio.1001150**

